# Magnitude of child sexual abuse and its associated factors among high school female students in Dire Dawa, Eastern Ethiopia: a cross-sectional study

**DOI:** 10.1186/s12978-021-01277-7

**Published:** 2021-11-10

**Authors:** Legesse Abera, Abdulahi Aliye, Kalbesse Tadesse, Alemu Guta

**Affiliations:** 1 Department of Midwifery College of Medicine and Health Sciences, Dire Dawa University, Dire Dawa, Ethiopia; 2 Department of Public Health College of Medicine and Health Sciences, Dire Dawa University, Dire Dawa, Ethiopia; 3 Department of Psychology College of Social Sciences, Dire Dawa University, Dire Dawa, Ethiopia

**Keywords:** Child, Sexual abuse, High school, Female students, Dire Dawa Administration

## Abstract

**Background:**

Child sexual abuse (CSA) refers to the involvement of a child (< 18 years) in sexual activity that he or she does not fully comprehend, is unable to give informed consent to, or for which the child is not developmentally prepared and cannot give consent, or that violates the laws or social taboos of society. It is a serious public health problem affecting millions of people each year worldwide but the most neglected and least documented form of violence in Ethiopia among school girls. Therefore, this study aimed to assess the magnitude of child sexual abuse and its associated factors among female high school students in the Dire Dawa administration, Eastern Ethiopia.

**Methods:**

An institutional-based cross-sectional study was conducted among female high school students in Dire Dawa administration between March 1 and 23/2021. We employed stratified and multistage sampling techniques to include 794 participants from selected high schools. A pretested self-administered questionnaire was used, and the data were analyzed using the SPSS software version 24.

**Results:**

The proportion of students who reported at least one form of sexual abuse was 384 (48.9%) and approximately 19% of the students reported rape from the total respondents. Students who live alone 4.3 times (AOR 4.30; 95% CI 1.81–10.24), those who live with their friends five times (AOR 5.02; 95% CI 2.24–8.04), and those who live with their single parent three times (AOR 3.31; 95% CI 1.23–8.89) more likely to experience sexual abuse than those living with both parents. The odds of experiencing sexual abuse among students of rural residence were 3.2 times higher than their urban counterparts (AOR 3.2; 95% CI 2.02–4.51). Students who didn’t drink alcohol were 70% more protective than those who drank alcohol (AOR 0.70; 95% CI 0.28–0.97). Among rape survivors (64, 37.9%) developed unwanted pregnancies, 26.0% of them underwent an abortion, and (39, 26.0%) developed STI as an outcome of sexual abuse.

**Conclusion:**

This study demonstrated that the magnitude of child sexual abuse among female students in Dire Dawa was high. Lack of discussion about sexual issues with parents, living without both parents, drinking alcohol, and being a rural residence had a significant association with child sexual abuse. Unwanted pregnancy, abortion, and STIs have been reported as reproductive health outcomes of rape. Therefore, policymakers should introduce and strengthen comprehensive sexual and reproductive health education both in school and out of school, in addition to formal education to reduce the magnitude of the problem. Parents should discuss all sexual and reproductive health issues with their children to reduce the magnitude and consequences of child sexual abuse.

## Plain language summary

Child sexual abuse (CSA) refers to the involvement of a child (< 18 years) in sexual activity that he or she does not fully comprehend, is unable to give informed consent to, or for which the child is not developmentally prepared and cannot give consent, or that violates the laws or social taboos of society.” Child sexual abuse is a common and serious public health problem affecting millions of people each year worldwide but the most neglected and least documented form of violence in Ethiopia among school girls. This study aimed to assess the magnitude of child sexual abuse and its associated factors among female high school students in the Dire Dawa administration, Eastern Ethiopia. In this study, an institutional-based cross-sectional study design was conducted among female high school students in the Dire Dawa administration between March 1 and 23/2021. Stratified and multistage sampling techniques were employed to include 794 participants from selected high schools. A pretested self-administered questionnaire was used and the data were analyzed using the SPSS software version 24.

The findings of this study showed that the proportion of female students who reported at least one form of sexual abuse was 384 (48.9%) and approximately 19% of female students reported rape from the total respondents. Students who live alone 4.3 times, those who live with their friends five times, and those who live with single parents three times more likely to experience lifetime sexual abuse than those living with both parents. The likelihood of experiencing lifetime sexual abuse among students of rural residence was 3.2 times higher than their urban counterparts. Students who didn’t drink alcohol were 70% more protective than those who drank alcohol.

In conclusion: this study demonstrated that the magnitude of child sexual abuse among female students in the Dire Dawa administration is high. Lack of discussion about sexual issues with parents, living without both parents, drinking alcohol, and being a rural residence had a significant association with child sexual abuse. Unwanted pregnancy, abortion, and STIs were the most commonly reported reproductive health consequences of sexual abuse. Therefore, policymakers should introduce and strengthen comprehensive sexual and reproductive health education both in school and out of school, in addition to formal education to reduce the magnitude of the problem. Parents should discuss all sexual and reproductive health issues with their children to reduce the magnitude and consequences of child sexual abuse.

## Background

Child sexual abuse (CSA) refers to the involvement of a child (< 18 years) in sexual activity that he or she does not fully comprehend, is unable to give informed consent to, or for which the child is not developmentally prepared and cannot give consent, or that violates the laws or social taboos of society [1]. It is one of the most common human rights violations and is now recognized as a public health priority. Sexual abuse affects people of all genders, sexual orientations, and ages in every community but the majority of victims are children, adolescents, and women in both industrial and developing countries [2].

Globally, one in three adolescent girls’ reports had their first sexual experience as a result of coercion. Sexual abuse is common in Sub-Saharan African educational institutions including Ethiopia. School adolescents can be victimized at school which may be verbal harassment or physical nature such as unwanted touching. It can also be more overtly violent as in cases where girls are sexually assaulted (raped) in or near school premises [3].

CSA is the least documented form of violence in many developing countries including Ethiopia, and only 1 in 10 incidents have been reported [4]. Factors such as inconsistencies in the definitions of sexual abuse, fear and social stigma against rape survivors, committed in complete secrecy, and social and cultural norms are contributed to underreporting [5].

Studies have shown that sexual violence against girls by older male students and teachers is very common and more than 40% of school girls have experienced some form of sexual abuse at some point in their lives. This leads to lower girls’ educational attainment and increases absenteeism and dropout rates [4, 6].

The WHO study found that 0.3 to 12% of female respondents reported being forced to have sex that they did not want to since the age of 15 years. Peru, Samoa, and the United Republic of Tanzania reported the highest levels from 10 to 12% [7]. A study conducted among female University Students in Northern Nigeria showed that the prevalence of violence was 58.8%. Of these 22.2% experienced sexual abuse and 50.8% experienced verbal abuse [8].

A systematic review in Ethiopia showed that the lifetime prevalence of sexual abuse against women by husbands or intimate partners ranged from 19.2 to 59% [9]. Another study conducted on sexual abuse in Debarik among high school female students also showed that sexual abuse is still a common phenomenon among young girls. The prevalence of performed and attempted rape was 8.8% and 11.5%, respectively [10].

Sexual abuse is strongly linked to social determinants such as poor governance, weak rule of law, cultural, social, and gender norms, unemployment, low income, gender inequality, and limited educational opportunities [11]. Factors such as the absence of one or both parents or being raised by a stepfather, parental conflicts, family adversity, lack of parental control and social isolation have also been linked to a higher risk of child sexual abuse [12].

Most of the reproductive health consequences of sexual abuse include: HIV/AIDS, unwanted pregnancy, unsafe abortion, sexually transmitted diseases, tears, and bleeding. Rape alone results in approximately 32,000 unwanted pregnancies each year. The consequences of CSA affect not only the victim but also their families’ future relationships and society as a whole. Therefore, CSA is a complex societal problem that requires a comprehensive response [3, 13, 14].

In Ethiopia, scientifically documented information regarding child sexual abuse is scarce among adolescents. Little has been explored about the magnitude of different types of child sexual abuse among high school students. To the best of our knowledge no studies related to CSA have been conducted by the Dire Dawa Administration. Although different studies have been conducted in different parts of the country with similar settings or contexts the existing studies are very old which is why we want to determine the current magnitude of the problem. Based on this understanding this study aimed to assess the magnitude of child sexual abuse and its associated factors among female high school students in Dire Dawa administration, Eastern Ethiopia.

## Methods

### Study design and setting

This study employed an institutional-based cross-sectional study design from March 1 to 20/2021 among 794 high school female students in the Dire Dawa Administration (DDA). DDA is located in the Eastern part of Ethiopia and is bordered by the regions of Oromia and Somali. The DDA Council consists of the city of Dire Dawa and the surrounding rural areas. Dire Dawa is an ancient city in Ethiopia found around 515 km from Addis Ababa (the capital city of Ethiopia). According to 2019 population projections, almost 0.5% of the Ethiopian population lives in the DDA and 10% of its population are children under 5 years of age. A total of 313,000 (63.5%) people lived in Dire Dawa City and 180,000 (36.5%) lived in rural areas. The total fertility rate is 3.1 for women of reproductive age (15–49 years) [15].

The potential health service coverage of the Dire Dawa administration was 89% with two governmental hospitals, 15 health centers, and 34 health posts. There are also 2 TVET Schools, 5 private colleges, 1 University, and 25 high schools (22 urban and 3 rural) high schools with a total number of students of 15,839 (8503 males and 7336 females) [16].

According to the education statistics annual abstract (ESAA) 2018/2019 the school dropout for grades 1–8 in 2017/2018 was 10.6% while the repetition rate was 7.9% in Dire Dawa but no data for high school students. The proportion of child marriage and teenage childbearing in Dire Dawa was 32% and 12.5% respectively [15, 17]. The proportion of high school students’ abortions was 6.9% [18].

### Study participants

The Source population for this study was all high school female students attending their education in the academic year 2020/2021 in DDA and the study participants were high school female students randomly selected from the source population and met the inclusion and exclusion criteria. Critically ill students, night and weekend students were excluded from the study because of invisibility in obtaining the data. Students who met the criteria but didn’t consent to participate in the study were also excluded.

### Sampling method and sample size determination

**Fig. 1:**
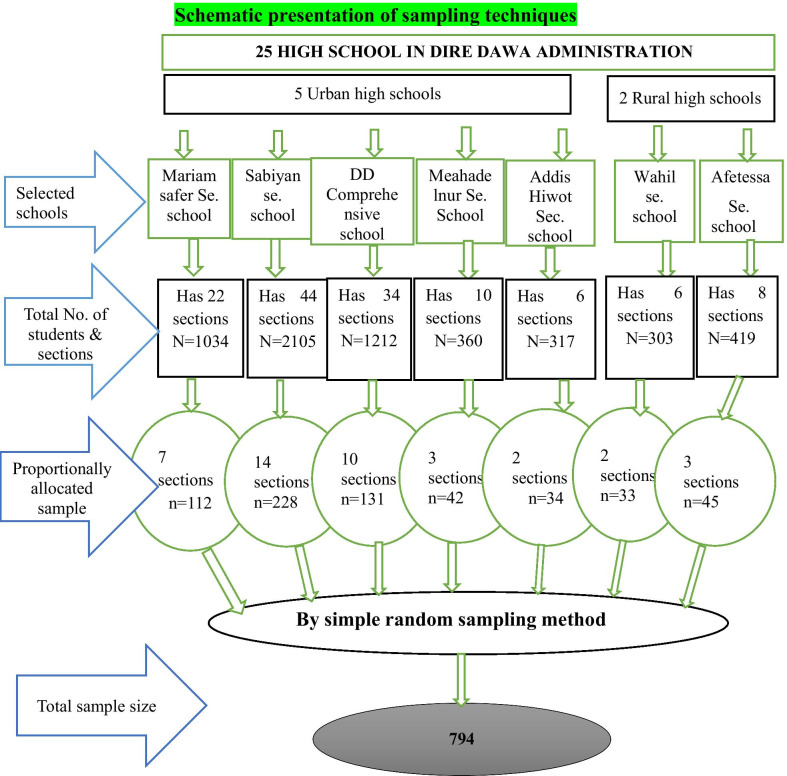
A schematic presentation of sampling on the study of child sexual abuse and its associated factors among high school female students in Dire Dawa administration, Eastern Ethiopia, March 1–23/2021

Stratified and multistage sampling techniques were employed in this study. First, we stratified high schools in the DDA into urban and rural areas. There were 25 high schools (22 urban and 3 rural) in the DDA. Five schools from urban and 2 rural schools were selected using the lottery method. A list of female students in each grade (9th–12th) and sections were taken from all the selected schools. A total of 40 sections from 130 sections were selected from the selected schools and the total sample size was proportionally allocated to the seven selected schools. Finally, by simple random sampling technique participants from all selected schools and sections were included in the study (Fig. 1). A total of 794 samples were determined using a single population proportion formula considering P = 45.4% [19, 20] 95% CI 5% margin of error, design effect 2, and 10% non-response rate.

### Data collection tools

A pre-tested structured and self-administered questionnaires were used as a data collection instrument which was partly adapted from the standard “childhood experience of care and abuse Questionnaire (CECA.Q)” [21]. The tools were first prepared in English and then translated into local languages and then back translations were made to check the consistency of the questionnaires. Pre-testing of the questionnaire was undertaken in 5% (40) female students in another high school before the actual data collection took place and corrections on the instrument were made accordingly.

### Data collectors and data collection procedures

Seven female nurses were recruited as data collectors and facilitators. Data collectors and facilitators were selected based on their previous experiences with data collection and their ability to speak local languages. Two days of detailed training was provided for data collectors and facilitators on the aim of the study, data collection tools and methods of data collection. All authors supervised the data collection process. Data were checked for completeness, clarity, and consistency by the facilitators and investigators as soon as they were collected.

To maintain confidentiality a separate room for students was arranged ahead and the data collectors and facilitators were assigned to each room. All selected participants were called and made to sit in the previously arranged rooms. Each student took a single seat with a sparse arrangement of chairs and desks and a face mask was distributed to all participants before providing the questionnaire to prevent COVID-19. Study participants were instructed not to include their names or other identifiers on the questionnaire and to fill out the questionnaire and leave it in the prepared collecting box. This was followed by the awareness creation class with regard to CSA.

### Data analysis

Data were cleaned, edited, coded, and entered into Epi-data version 3.1 software then exported to SPSS software version 24.0 for analysis. Descriptive statistics such as frequencies, proportions, and summary statistics were used to describe the study population with relevant variables. Binary Logistic regression was used to assess the presence of the association between dependent and independent variables and variables with p-value < 0.25 were as candidates for multivariable logistic regression. Adjusted odds ratios, P-value < 0.05 with 95% CI were used to determine the significance and level of association between the dependent and independent variables.

### Data quality control

To ensure data quality the questionnaire was translated into local languages and back-translated into English to check for consistency. Pre-testing of the questionnaire was undertaken in 5% (40) female students in Lagahare high school before the actual data collection took place and corrections on the instrument were made accordingly. Two-day training was provided for data collectors and facilitators and all authors supervised the study closely. Data were checked for completeness, clarity, and consistency by the facilitators and investigators as soon as they were collected. Finally, data were entered through double data entry into the software Epi-data version 3.1 to minimize error.

### Study variables

- Dependent variable:
  - Child sexual abuse experience (Yes/No)
- Independent variables
  - Socio-demographic factors
    Grade, Age, Marital status, educational status
    Religion, Residence, Ethnicity, average monthly income
  - Family level factors
    Family income, parent educational level, Family size,
    Parent’s occupation, living with a stepfather, Living arrangement, absence of parents
  - Individual factors
    Living out of home, living alone, Age at first sexual practice, having boyfriend,
    SRH open discussion with parents
  - Peer and behavioral factors
    Peer pressure, Smoking, Drinking, Chewing

### Operational definition

- Child sexual abuse (CSA): refers to the involvement of a child (< 18 years) in sexual activity that he or she does not fully comprehend, is unable to give informed consent to, or for which the child is not developmentally prepared and cannot give consent, or that violates the laws or social taboos of society” [1].
- Child sexual abuse experience: This means in this study, female students self-reported involvement in any sexual activity in her lifetime that she did not fully comprehend and was unable to give informed consent to or forcefully sexual activities.
- The physical forms of sexual abuse include: fondling, oral-genital contact, rape or attempted rape, and use of a child for pornography [22].
- Non-physical forms of sexual abuse include indecent exposure, plain talk about sex designed to shock a child or arouse her curiosity, allowing the child to watch or hear sexual acts or materials, and having sex in front of the child [22].
- Rape: a type of sexual assault usually involves sexual intercourse or other forms of sexual penetration carried out against a person without the consent of the person.
- Substance abuse: the use of a drug in amounts or by methods that are harmful to the individual or others.
- Incest: human sexual activity between family members or close relatives (blood relations) [23].

## Results

### Socio-demographic characteristics of respondents

A total of 785 female students completed the questionnaire appropriately and gave a response rate of 98.8%. More than half (52.1%) of the participants were between the age of 16–17 years with a mean and standard deviation of 16.0 ± 1.5 years. Most participants were from grade 9 (27.3%) and grade 10 (26.0%) and grade 12 (21.7%).

Regarding religion (40.0%) of the respondents were Muslim and 35.0% were Orthodox Christians. Oromo accounts (42.5%) followed by Somali (35.0%) in Ethnicity and 95% of the participants were single. Among the participants (67%) were from urban residences while the rest (33%) were rural. Half (50.0%) of the respondent's father's educational status was secondary education and below. Moreover, 31.8% of the participants' maternal education was above secondary school. A large proportion (60.0%) of participant’s fathers and 33.5% of their mothers were employees but nearly half (48.9%) of respondent's mothers were housewives. The living arrangements of the students indicated that only 45.2% of the respondents were living with both parents (Table 1).

**Table 1:** Socio-demographic characteristics of respondents on the study of magnitude of CSA and its associated factors among high school female students in DDA, Eastern Ethiopia, March 2021

Variables	Alternative response	Number	%
Age of respondents	14–15	93	11.8
	16–17	409	52.1
	18–19	172	21.9
	≥ 20	111	14.1
Marital status of respondents	Single	746	95.0
	Married	39	5.0
Respondents living condition (arrangement)?	Both parents	355	45.2
	Single parent	192	24.5
	Friends	118	15.0
	Alone	120	15.3
With whom you slept together in your home?	Mother	197	25.1
	Sister/s	393	50.1
	Brother/s	80	10.2
	Alone	115	14.6
What is your father’s occupation?	Employee	471	60.0
	Merchant	114	14.5
	Farmer	80	10.2
	Daily labourer	120	15.3
What is your mother’s educational status?	Grade 1–4	137	17.5
	Grade 5–8	198	25.2
	Grade 9–12	200	25.5
	Above grade 12	250	31.8
Who support you for learning?	Parents	355	45.2
	Siblings	192	24.5
	Relatives	118	15.0
	Husband/boyfriends	120	15.3
Your family sizes	< 5	378	48.1
	5 and above	407	51.9

### Respondents substance utilization status

Concerning substance utilization 118 (15.0%) respondents chewed Khat, 39 (5.0%) smoked cigarettes or tobacco and 90 (11.5%) drank alcohol in their lifetime. Moreover, 115 (14.6%) and 78 (10.0%) participants reported that they were currently chewing khat and drinking alcohol respectively but none of them smoked. Similarly, 198 (25.2%) of the respondents reported that they had either male or female friends who drank alcohol or chewing khat or both (Table 2).

**Table 2:** Substance utilization status of respondents on the study of magnitude of Child Sexual Abuse and its associated factors among high school female students in Dire Dawa Administration, Eastern Ethiopia, March 2021

Variables	Alternative response	Number	%
Have you ever chewed Khat?	Yes	118	15.0
	No	667	85.0
Are you chewing currently?	Yes	115	14.6
	No	670	85.4
Chat chewing frequency (n = 115)	Once in a week	37	32.2
	Twice a week	46	40.0
	Once in a month	5	4.3
	Twice in a month	27	23.5
Have you ever drunk alcohol?	Yes	90	11.5
	No	695	88.5
Are you drinking currently? (n = 90)	Yes	78	86.7
	No	12	13.3
Alcohol drinking frequency (n = 78)	Once in a week	16	20.5
	Twice a week	38	48.7
	Once in a month	12	15.4
	Twice in a month	12	15.4
Do your friend drink alcohol (chewing) or both?	Yes	198	25.2
	No	587	74.8

### Sexual and reproductive history of respondents

In this study, 279 (35.5%) participants had ever had a boyfriend and 181(23.0%) reported having a history of sexual intercourse. The mean age at first sexual practice was 15 years with ± 1.5 SD. Of those who had started sexual intercourse (150, 82.9%) started sexual intercourse without their willingness and only 31 (17.1%) of them started sexual intercourse with their will. Almost half (50.3%) of respondents who started sex had multiple (two or more) lifetime sexual partners. Four hundred eight (52.0%) of the study subjects had open discussions with their parents about sexual and reproductive health issues while the rest didn’t (Table 3).

**Table 3:** History of child sexual abuse experiences among high school female students in Dire Dawa administration, Eastern Ethiopia, March, 2021

Variables	Alternative response	Number	%
Have you ever had boyfriend?	Yes	279	35.5
	No	506	64.5
How many boyfriends did you have in your life?	Only one	139	49.8
	Two or more	140	50.2
Have you ever had history of sexual intercourse?	Yes	181	23.0
	No	604	77.0
Was sexual intercourse based on your will? (n = 181)	Yes	31	17.1
	No	150	82.9
What was your age at which you started sex? (n = 181)	10–13 year	39	21.5
	14–17 year	95	52.5
	≥ 18 year	47	26.0
Have you had any discussion with your parents about SRH?	Yes	408	52.0
	No	377	48.0

### Magnitude and types of sexual abuse respondents experienced

**Fig. 2:**
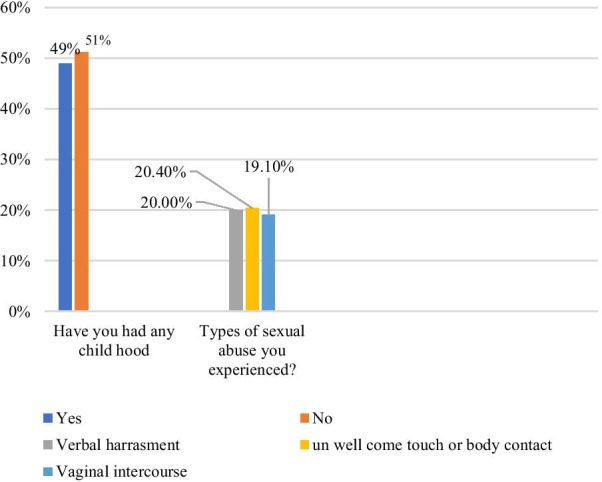
Magnitude and types of sexual abuse that the respondents ever experienced (encountered) in their life time among high school female students in Dire Dawa administration, Eastern Ethiopia, March 2021

To assess lifetime CSA participants were asked to report on their experiences of abuse during their entire childhood and adolescence. The proportion of female students who reported at least one form of sexual abuse was 384 (48.9%) and the proportion of female students who reported rape from the total respondents was approximately 150 (19%). Concerning the types of sexual abuse, respondents experienced most, 20.4% (95% CI 20.5–30.7) of participants were reported verbal form of sexual abuse (verbal harassment), 20.4% (95% CI 20.5–30.7) of them reported unwelcomed touch/body contact, 19.1% (95% CI 18.3, 23.7) reported rape. Moreover, 20% of any form of sexual abuse was experienced in this academic year, 16% experienced it in the last academic year and 12.9% experienced it before 3 years (Fig. 2).

The majority (93, 62.0%) of rape survivors were raped at the age of less than 15 years and 75 (50.0%) of the rape survivors were living alone, 23.3% lived with their friends, and 20.0% lived with their single parent when they became raped. The majority (29.3%) of rape took place in the survivors’ homes and 24.7% in the perpetrator's home, 17.3% took place in the Hotel, 22.7% took place inside the car, and the rest 6.0% in the public street (field).

Moreover, the majority (76.0%) of the rape victims didn’t report the case to anybody and only (36, 24.0%) disclosed their case to someone else. Twenty (13.3%) reported to their friends, 10(6.7%) reported to legal bodies, and 6(4.0%) of them were reported to their parents. The main reasons for not reporting the incidence were 34(29.8%) because of fear of perpetrators, 22(19.3%) because of fear of their families, 30(30.7%) because of fear of stigma, and the remaining 23(20.2%) of them didn’t know what to do.

### Perpetrator’s characteristics

Most perpetrators (abusers) were peers, non-relatives, relatives, and strangers. Among 150 rape survivors (125, 83.3%) were raped by extra-familial members, and the remaining (25, 16.7%) were raped by family members. Commonly reported extra-familial perpetrators were peers (schoolmates) (21.2%), school teachers (20.8%), boyfriends (19.8%), neighbors (16.6%), and unrecognized persons (strangers) (4.9%).

### Reproductive health consequences of child sexual abuse (rape)

**Fig. 3:**
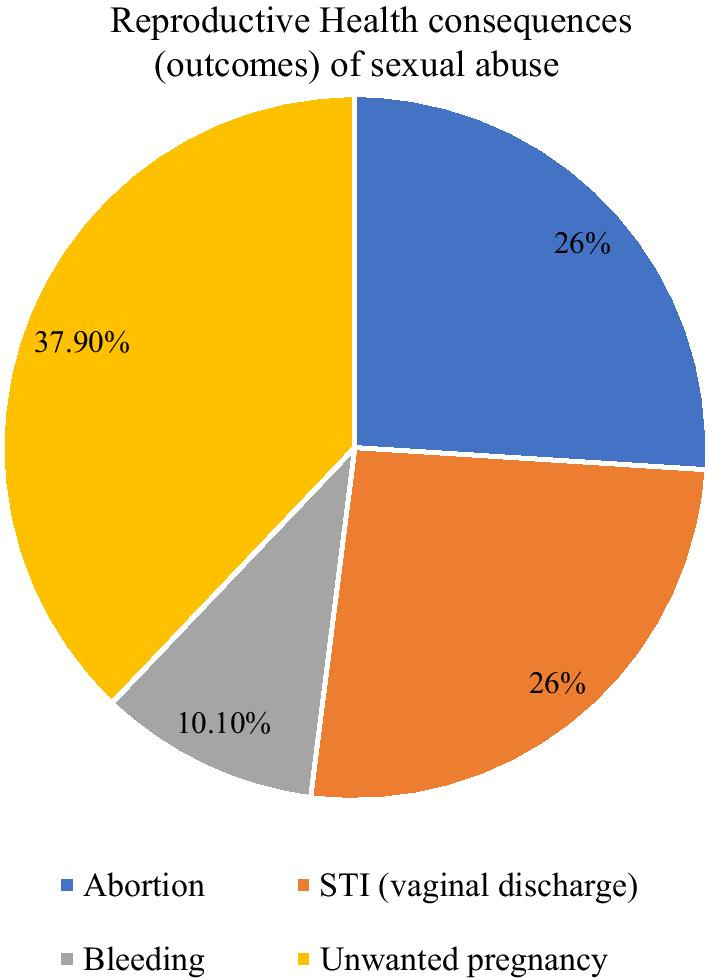
Reproductive health consequences of sexual abuse for the study of child sexual abuse and its associated factors among high school female students in Dire Dawa administration, Eastern Ethiopia, March, 2021

Among the total (150) rape survivors (64, 37.9%) developed unwanted pregnancies and 26.0% underwent an abortion. Others also developed STI (vaginal discharge) (39, 26.0%) and 10.1% had vaginal bleeding immediately after being raped (Fig. 3).

### Factors associated with child sexual abuse

Factors associated with child sexual abuse were assessed in this study. In bivariate analysis students living arrangement (condition), having parent-adolescent communication about sexual and reproductive health, father’s educational level, residence, substance use, having a friend who drinks alcohol, and family monthly average income had a statistically significant association with child sexual abuse. Variables with p < 0.25 in the bivariate analysis, were selected for multivariate logistic regression. In multivariate logistic regression after controlling for potentially confounding variables: living arrangement (condition), having an open discussion (communication) with parents on sexual and reproductive health, residence, and substance use had a statistically significant association with child sexual abuse.

Students’ living arrangements (conditions) were associated with the occurrence of child sexual abuse. Female students living alone 4.3 times (AOR 4.30; 95% CI 1.81–10.24), those living with their friends five times (AOR 5.02; 95% CI 2.24–8.04), and those living with their single parent three times (AOR 3.31; 95% CI 1.23–8.89) more likely experience sexual abuse than those living with their both parents.

Participants who did not have an open discussion with their parents on sexual and reproductive health were about three times more likely to experience sexual abuse than those students who had an open discussion with their parents (AOR 2.93; 95% CI 1.33–6.45).

The odds of experiencing lifetime sexual abuse among students of rural residence were 3.2 times higher than their urban counterparts (AOR 3.2; 95% CI 2.02–4.51). Students who didn’t drink alcohol were 70% more protective than those who drank alcohol (AOR 0.70; 95% CI 0.28–0.97) (Table 4).

**Table 4:** Factors associated with child sexual abuse among high school female students in Dire Dawa administration, Eastern Ethiopia, March, 2021 (384)

Variables	Life time CSA		COR (95%CI)	COR (95%CI)
	Yes	No		
Respondents age category				
14–15 years	84	9	1.00	1.00
16–17 years	159	250	0.40 (0.92–2.95)	1.86 (0.52–2.05)
18–19 years	90	82	1.80(1.19–7.52)	1.20(0.19–5.54)
≥ 20 years	51	60	0.53 (0.43–0.99)	0.33 (0.12–1.93)
Marital status of respondents			2.0	
Married	7	32	1.00	1.00
Single	377	369	3.0(1.135–3.726)	2.156(0.674–6.89)
Father’s educational status				
Above secondary	142	251	1.00	1.00
Below secondary	242	150	5.59(2.28–13.9)	4.69(0.84–11.95)
Living arrangement with				
Both parents	76	90	1.00	1.00
Single parent	90	93	3.00(1.19–7.54)	3.31(1.23–8.89)*
Friends	192	198	3.01(1.14–7.41)	5.02(2.24–8.04)*
Alone	26	20	2.44(1.21–7.11)	4.30(1.81–10.24)*
Have you open discussion on SRH?				
Yes	154	254	1.00	1.00
No	230	147	3.17(1.53–6.58)	2.93(1.33–6.45)*
Family monthly income				
< 5000 ETB	200	156	1.00	1.00
5000–9999 ETB	105	95	5.08(2.44–10.55)	3.82(0.76–8.31)
10,000–15,000 ETB	67	90	2.5(1.22–7.08)	0.42(0.92–6.98)
> 15,000 ETB	12	60	0.53(0.22–0.99)	0.50(0.08–0.87)
Residence of respondents				
Urban	225	301	1.00	1.00
Rural	159	100	2.0 (1.02–3.05)	3.2(2.02–4.51)*
Substance use (drink alcohol)				
Yes	333	180	1.00	1.00
No	51	221	0.53(0.22–0.99)	0.70(0.28–0.97)*
Having friend who drink alcohol				
Yes	148	50	1.00	1.00
No	236	351	0.32(0.12–0.79)	0.50(0.08–0.87)*

*COR* crude odds ratio, *AOR* adjusted odds ratio

“*” has significant association

## Discussion

Childhood sexual abuse harms the adulthood of individuals who face the incident. The magnitude of sexual abuse among children varies substantially in different studies [24]. This study revealed that a significant proportion (384, 48.9%) of female students reported experiencing at least one form of sexual abuse in their lifetime.

This finding is consistent with studies conducted in Walayita Sodo, Ethiopia 45.4% [19], Mekelle town, Ethiopia 45.4% [20], Bahir Dar city, Ethiopia, 49.1% [25], India, 47.0% [26], southwest Nigeria 42.1% [27], and Japan 52.5% [28]. However, the result of this study was much higher than those of a study conducted among high school adolescents in Addis Ababa, Ethiopia 12.7% [29], Harari Regional State, Ethiopia 25% [30], and Butajira, southern Ethiopia 32.8% [31]. However, the results were lower than those of studies conducted in Southwest Ethiopia, 68.7% [32], and southern Brazil 56% [33].

These discrepancies may be due to social and cultural differences between these study subjects in reporting sexual abuse such as fear of stigma, lack of knowledge about sexual abuse, deference at the time of the study, and could be the socio-demographic differences of the study populations.

The finding of this study again demonstrated that the proportion of female students who reported rape from the total respondents was about 19% (95% CI 18.3–23.7). This finding is similar to the studies conducted in Bahir Dar city, Ethiopia 16.7% [25], and in Addis Ababa, 23% [29]. However, the study results were higher than those of studies conducted in Butajira, 6.3% [31], in Arbaminch town, 11.0% [34], and in Debark, Ethiopia, 8.8% [10]. These discrepancies may be due to social and cultural differences between the study subjects in reporting sexual abuse such as fear of stigma, lack of knowledge about sexual abuse, due to differences in the time of the study, and could also be the socio-demographic differences in the study populations.

Parent-adolescent communication on sexual and reproductive health-related issues is crucial for adolescents to learn and share life experiences on SRH from families which helps them to prevent risky sexual behavior. The findings of this study revealed that female students who had no open discussions with their parents about SRH were about three times more likely to experience sexual abuse than those students who had open discussions with their parents.

This finding is consistent with a study conducted in Bahir Dar town, Ethiopia, and Arbaminch town, Ethiopia where the odds of experiencing lifetime rape were much higher among students who never had open discussions with parents on SRH than among those who had it [25, 34]. This might be because most students and families consider open discussions about sexual issues as shame and taboo in Ethiopian cultures, resulting in reluctance and fear of discussing and addressing sexual health issues. This leads to missing opportunities for parents to acquire experiences and life skills in the prevention of sexual abuse.

Again, our study showed that female students living alone 4.3 times, those living with their friends five times, and those living with their single parents three times more likely to experience sexual abuse than those living with both parents. These findings are in line with the studies done among high school students in Arbaminch town, Ethiopia, in Harar, and southeast Nigeria [27, 30, 34]. The possible explanation could be children living with their parents are under direct monitoring and follow up and the parents care for their daughter more than their friends and relatives this can minimize their chance of exposure to sexual abuse.

Some studies done in Ethiopia underlined the use of alcohol as contributing factor for sexual abuse [9]. Similarly, the finding of this study revealed that respondents who had a history of alcohol consumption and those having a peer or friend drink alcohol at higher risk of experiencing sexual abuse than their counterparts. Students who don’t drink alcohol were 70% less likely to experience sexual abuse than students who drink alcohol (AOR 0.70; 95%CI 0.28–0.97). Moreover, the odds of experiencing sexual abuse were two times higher for those respondents who had a close friend drinking alcohol (AOR 2.0; 95%CI 1.09–5.43) than their counterparts. This finding is also supported by the study conducted in Bahirdar town, Ethiopia [25]. This could be explained by the fact that alcohol leads to a reduction in the decision-making ability of an individual on her sexual and reproductive health matters.

Some study findings show that socio-economic characteristics have nothing to do with child sexual abuse. They found no significant link between child sexual abuse and socioeconomic backgrounds such as father's occupation, residence, and average family monthly income [35].

Contrary to this our study showed that rural residence was strongly associated with child sexual abuse. Students from rural residences were 3.2 times more likely to experience sexual abuse than those students from urban residences (AOR 3.2; 95% CI 3.02–4.51). Similarly, studies were conducted in Bahir Dar city, Ethiopia [25]. The possible explanation could be students from urban relatively have better access to information through youth associations, youth centers, the media, and the environment itself. However, their counterparts from rural areas might lack such chances because of low awareness of the society which inhibits free and open discussion about reproductive and sexual issues.

The most frequently reported reproductive health consequences as a result of sexual abuse (rape) in this study were: unwanted pregnancy 64 (37.9%), abortion (26.0%), STI (vaginal discharge) (26.0%), and vaginal bleeding (10.1%). This finding is similar to the study conducted in Harar which showed that unwanted pregnancy (44.2%), abortion (32.2%), vaginal discharge (28.6%), and genital trauma (25%), were the most common consequences of rape [30]. Again, our study result was similar to the studies conducted in Addis Ababa and Debark town [10, 29], which showed consequences of forced sex as reported by the respondents were: injury around the genitalia (33.3%), unusual vaginal bleeding (20%), pregnancy (16.7%), and swelling around the genitalia (13.3%) [18, 35].

## Limitations of the study

Since our study topic assesses personal and sensitive issues related to sexuality, this might have caused underreporting of experiences of sexual abuse. This study was an institutional-based study that might not be representing the wider community (out-of-school female adolescents). Because of the cross-sectional nature of our study, the cause and effect relationship could not be ascertained.

Another important limitation of this study could be the lack of a globally acceptable definition of CSA and acts that constitute CSA. Thus, the findings of this study should be interpreted within these limitations.

## Conclusion

This study demonstrated that child sexual abuse is still a common problem among high school female students in the Dire Dawa Administration. Almost half of the female students have experienced lifetime at least one form of sexual abuse. Lack of discussion about sexual and reproductive health issues with parents, living without both parents, drinking alcohol, and being a rural residence had a significant association with child sexual abuse. The most common reproductive health consequences of sexual abuse in this study were unwanted pregnancy, abortion, and STIs.

## Implications

Policymakers should introduce and strengthen comprehensive sexual and reproductive health education both in the school and out-of-the-school besides formal education to reduce the magnitude of the problem.

This study showed the importance of parent-adolescent communication about sexual and reproductive health. Therefore, parents should discuss all sexual and reproductive health issues with their children to reduce the magnitude and consequences of child sexual abuse.

Since students living without parents are at higher risk of experiencing CSA, parents should monitor and give due attention to female students. Female children should attend their school living with their family or responsible caretaker rather than left alone or with their friends to reduce the risk of victimization and increase prevention methods.

School-based awareness creation on prevention of common consequences of sexual abuse and those who experienced sexual abuse should be immediately identified and enrolled in school and/or community-based support programs.

More comprehensive nationwide research on child sexual abuse may bring out the whole picture of the issue assisting policymakers, preventive measures, and therapeutic interventions.

## Data Availability

The data sets used and/or analyzed during the current study are available from the corresponding author on reasonable request.

## Abbreviations

ASRH: Adolescent sexual and reproductive health
AIDS: Acquired immune-deficiency syndrome
CRC: Convention on the rights of the child
CSA: Child sexual abuse
CDC: Center for disease control
DDU: Dire Dawa University
HIV: Human Immune Deficiency Virus
SRH: Sexual and reproductive health
STDs: Sexual Transmitted Diseases
STI: Sexually Transmitted Infections
TVET: Technical Vocational Education Teaching
UNICEF: United Nations International Children’s Fund
WHO: World Health Organization
